# t^4^ report^1^: Supporting Read-Across Using Biological Data

**DOI:** 10.14573/altex.1601252

**Published:** 2016-02-11

**Authors:** Hao Zhu, Mounir Bouhifd, Nicole Kleinstreuer, E. Dinant Kroese, Zhichao Liu, Thomas Luechtefeld, David Pamies, Jie Shen, Volker Strauss, Shengde Wu, Thomas Hartung

**Affiliations:** 1 Department of Chemistry, Rutgers University, Camden, NJ, USA; Center for Computational and Integrative Biology, Rutgers University, Camden, NJ, USA; 2 Johns Hopkins Bloomberg School of Public Health, Center for Alternatives to Animal Testing (CAAT), Baltimore, MD, USA; 3 National Toxicology Program Interagency Center for the Evaluation of Alternative Toxicological Methods, National Institute of Environmental Health Sciences, Research Triangle Park, NC, USA; 4 Risk Analysis for Products in Development, TNO Zeist, The Netherlands; 5 US FDA, NCTR, Little Rock, Arkansas, USA; 6 Research Institute for Fragrance Materials, Inc. Woodcliff Lake, New Jersey, USA; 7 BASF Aktiengesellschaft, Experimental Toxicology and Ecology, Ludwigshafen, Germany; 8 Procter & Gamble, Cincinnati, OH, USA; 9 University of Konstanz, CAAT-Europe, Konstanz, Germany

**Keywords:** read-across, biological similarity, safety assessment, big data

## Abstract

Read-across, i.e. filling toxicological data gaps by relating to similar chemicals, for which test data are available, is usually done based on chemical similarity. Besides structure and physico-chemical properties, however, biological similarity based on biological data adds extra strength to this process. In the context of developing Good Read-Across Practice guidance, a number of case studies were evaluated to demonstrate the use of biological data to enrich read-across. In the simplest case, chemically similar substances also show similar test results in relevant *in vitro* assays. This is a well-established method for the read-across of e.g. genotoxicity assays. Larger datasets of biological and toxicological properties of hundreds and thousands of substances become increasingly available enabling big data approaches in read-across studies. Several case studies using various big data sources are described in this paper. An example is given for the US EPA’s ToxCast dataset allowing read-across for high quality uterotrophic assays for estrogenic endocrine disruption. Similarly, an example for REACH registration data enhancing read-across for acute toxicity studies is given. A different approach is taken using omics data to establish biological similarity: Examples are given for stem cell models *in vitro* and short-term repeated dose studies in rats *in vivo* to support read-across and category formation. These preliminary biological data-driven read-across studies highlight the road to the new generation of read-across approaches that can be applied in chemical safety assessment.

## 1 Introduction

Read-across has become a primary approach to fill data gaps for chemical safety assessments. Chemical similarity based on structure, reactivity and physico-chemical property information, is the main approach applied for this purpose. Chemical toxicity, however, normally contains complicated biological mechanisms, so only using chemical similarity to justify the read-across will induce errors, especially when the chemical similar compounds show dissimilar toxicity phenomenon. The availability of massive biological data for environmental compounds makes biological similarity approaches feasible within the scope of read-cross. This accompanying paper to the efforts toward Good Read-Across Practice (Ball et al., 2016, this issue) scopes the opportunities for biological support to strengthen read-across with a number of examples.

There are three different types of biological similarity approaches that have been applied: 1) When several bioassays represent key mechanisms of target toxicity endpoints, they may have straightforward predictive power and in some instances it has been possible to show this for a larger applicability domain by traditional validation. In some cases, biological similarity can be applied, however, successfully only for specific parts of the chemical universe and only one toxicity endpoint, which has been termed “local validity” (Patlewicz et al., 2014); 2) When biological similarity is based on a large number of bioassays, the read-across study may be successful for various types of toxicity endpoints. This kind of studies is so far rarely pursued because of expensive cost to screen the same target compounds against many (often several hundred) different bioassays (Zhu et al. 2014). The recent public availability of large High Throughput Screening (HTS) datasets (e.g. ToxCast and Tox21 data) has made this kind of study feasible. In this case, new statistical tools need to be applied for the read-across studies using complex biological data; 3) The concept of “toxicity pathways” (or the molecularly define pathways of toxicity, PoT (Kleensang et al., 2014)) represents a new opportunity of risk assessment. When all receptors within a potential pathway are included in the biological testing, the mechanism-based read-across studies are feasible. In the current big data era, some popular compounds, such as well-known toxicants, have been extensively studied worldwide and a complex data landscape is available for these compounds (e.g. omics data). To this end, more biological data is needed for target compounds, more toxicity mechanisms need to be clarified and novel computational tools (e.g. big data approaches) need to be developed. The recent efforts steered by OECD of developing Adverse Outcome Pathways (AOP) have added lots of useful information and tools regarding these needs (Vinken et al. 2013a; Vinken 2013b; Ankley et al. 2010). Biological similarity studies have been greatly enhanced by these rapidly increased biological data (Zhu et al. 2014).

Omics analyses allow also to assess similarity; here *in vivo* models, such as short-term animal studies as well as stem cell-derived developmental and organ models lend themselves for signatures of toxicity to be compared. While test-across deviates from traditional methods only by acknowledging the small applicability domain of proven usefulness, the HTS and omics approaches are based on what is now called “big data”, i.e. curated large datasets for data-mining.

## 2 The state of the art of read-across using biological data

### 2.1 Empirical read-across studies using biological data

#### 2.1.1. Moving from chemical structure information to biological data

The traditional read-across studies, mostly using Quantitative Structure-Activity Relationship (QSAR) approaches, were normally based on chemical structure information (Solimeo et al., 2012; Zhu et al., 2008; Schultz et al., 2003; Zhu et al., 2009). Certain structural fragments (e.g. structural alerts) (Klopman et al., 2004), physico-chemical properties (Klopman et al., 1999) or other molecular properties (e.g. molecular sizes) (Moss et al., 2002) were used to estimate the chemical toxicity potential. In contrast to these efforts, the early stage of using biological data in read-across normally uses limited biological data obtained from one or few bioassays for small sets of compounds.

In the studies using chemical information alone for large parts of the chemical universe, activity cliffs (i.e. small changes in structure inducing significant changes of toxicity) resulted in major prediction errors (Maggiora, 2006). For this reason, evaluation of certainty for read-across, as well as for *in silico* methods (Hartung and Hoffmann, 2009) and for *in vitro* assays, is crucial. Especially, the part of the chemical universe where a given method is applicable needs to be defined. This means that reliable predictions can be made within a certain applicability domain (Hartung et al., 2004). Without knowing all parts of the chemical universe to which a method is applicable, it is often possible to demonstrate that a method works for a certain group of chemicals. A new term, named “local validity”, was introduced to describe this issue in read-across studies (Patlewicz et al., 2014). When applying biological data in read-across, it should focus on the areas of local validity to carry out *in vitro* tests, which represent key aspects of the pathophysiology, a concept earlier introduced as test-across (Hartung, 2007). Because of the recent emergence of the public toxicity data from the European Registration, Evaluation, Authorisation and Restriction of Chemicals (REACH) and the US Tox21 datasets, which notably resulted in more than 1,700 overlap of chemicals (Luechtefeld et al., 2016a), this concept can now be empirically evaluated. These new efforts will help to move from a pragmatic use of weight-of-evidence to a quantitative biological data read-across with an associated measure of evaluation uncertainty (Linkov et al., 2015).

#### 2.1.2. Case study: developing bioassays for read-across evaluations of developmental toxicity

Substantial efforts have already been undertaken to develop alternative assays for the assessment of reproductive and development toxicity (Adler, et, al. 2011; Leist, et. al, 2014). Of these alternatives only a few have been formally validated for developmental toxicity, such as the whole embryo culture (WEC)^2^, the embryonic stem cells test (EST), and the mammalian micromass (MM) test (Pamies et al., 2011). While characterizing these assays, it was also recognized that none of them alone could cover the whole mammalian reproductive cycle due to its inherent complexity, covering male and female fertility, implantation, and embryonic development (Adler et al., 2011; Leist et al., 2014). Therefore, more recent studies have attempted to combine several *in vitro* assays into a test battery instead of applying individual assays. As part of a European FP6 project called ReProTect, a feasibility study was performed, in which ten compounds in a battery of 14 assays was studied (Schenk et al., 2010). This battery, which consisted of several assays detecting endocrine disruption (AR and ER receptor binding assays, and ARE and ERE promotor driven reporter assays), 3 tests detecting embryotoxicity (mEST, WEC, and ReProGlo), and several assays detecting adverse effects on male and female fertility (mouse follicle bioassay, bovine maturation and fertilization assays, mouse peri-implantation assay, and Ishikawa test). This battery was able to detect all reproductive toxicants, for which the modes of action were actually represented in at least one of the assays. In a subsequent study of the European FP7 project ChemScreen, this battery approach was given a follow-up (Piersma, et. al, 2013). The zebrafish embryo test (ZET) and the embryonic stem cell test (EST) were included as more apical assays, to detect effects on development of a whole egg from fertilization until the hatching stage 72 h later (Hermsen, et. al, 2011), and to detect effects on cellular differentiation of cardiomyocytes (Scholz, et. al, 1999), respectively. Again the ReProGlo assay, monitoring interference with the WNT pathway (Uibel, et. al, 2015), and assays for CYP17 and CYP19, to detect effects on steroidogenesis, enzymes essential for reproductive hormone homeostasis (van Duursen, et, al. 2010; Hecker, et. al, 2011) were included. Finally, a panel of 24 high-throughput CALUX assays were added to measure changes in activity of key transcription factors, varying from nuclear receptors (e.g. reproductive hormone receptors) to transcription factors involved in cellular signaling (Sonneveld et al., 2005; van der Burg et al., 2013). The approach also encompassed toxicokinetic modeling to reveal whether effective *in vitro* concentrations observed in the battery are in the range expected from the *in vivo* reproductive toxicity data, in line with suggestions by Daston et al. (2010). The ChemScreen battery approach (including the toxicokinetic model) successfully identified eleven out of twelve compounds with varying mechanisms of action, while the missed compound, glufosinate ammonium, had a mechanism not covered by the battery (Piersma et al., 2013). This result encourages to further optimize this battery into one ultimately able to detect all reprotoxic compounds.

The use and interpretation of battery results depends strongly on the purpose of testing and the information that may already be available. In the absence of any *in vivo* test information relevant to potential reproductive toxicity and/or in the absence of any structural alerts pointing to such effects, the battery could be applied as a filter optimizing and/or reducing the testing of potential reproductive toxicants in animal studies (Wu et al., 2013). The battery could also help for prioritizing chemicals for further investigation and/or by selecting candidate compounds (e.g. drug candidates) for further development (van der Burg et al., 2011). On the other hand, when there are clear indications for potential reproductive toxicity based on close structural similarity of a query chemical to a reproductive toxicant, the battery could be used to confirm any reproductive toxicity, and to avoid any further *in vivo* studies. Ideally, this battery should then also be capable to correctly distinguish reproductive toxicants from non-reproductive toxicants, even when both have high structural similarity. This has been investigated by Kroese et al. (2015) via testing three groups of structurally-related chemicals, differing in their reproductive toxicity: two valproic acid (VPA) analogs, i.e. 2-ethylhexanoic acid (EHA), and 2-methylhexanoic acid (MHA), two analogs of monoethylhexyl phthalate (MEHP), i.e. monobenzyl phthalate (MBzP), and monomethyl phthalate MMP), and three organotin analogs, i.e. tributyltin chloride, dibutyltin dichloride (TBTC), dibutyltin chloride dibutyltin dichloride (DBTC) and monobutyltin trichloride (MBTC). MHA and MMP were not considered as reproductive toxicants, while MBTC was considered as a weak reproductive toxicant. The battery correctly distinguished MMP and MBTC as non- or weak developmental toxicants. MHA was identified as a weak developmental toxicant by this battery, while it is negative in *in vivo* studies. However, available toxicokinetic data for MHA show clearly lower predicted fetal concentrations as compared to that of VPA and EHA, and at these lower concentrations the battery showed no developmental toxicity for MHA. This result clearly shows the relevance of toxicokinetic information for any assessment based on alternative *in vitro* models (Bosgra and Westerhout, 2015). Such *in vitro* batteries can be combined with *in silico* tools, bioassays in lower species or even short-term *in vivo* tests. An important improvement lies in combining these batteries of tests with algorithms for optimizing the employment and interpretation of the different components. Such Integrated Testing Strategies (Hartung et al., 2013; Rovida et al., 2015) hold promise for the *in vitro* prediction of complex endpoints. However, they represent enormous challenges for validation of such a battery. Before such validation efforts are actually possible, already test-across approaches for groups of chemicals similar to the examples given above could be carried out, where non-tested compounds are profiled together with chemically similar tested ones. Besides the above efforts, other complex bioassays representing broad biological processes possibly disturbed by a given chemical can serve to profile substances. The devTOX quickPredict (devTOXqP) assay was developed around the principal that toxicity is a function of exposure. The assay uses human embryonic or induced pluripotent stem (hPS) cells to predict a test article’s developmental toxicity potential based on changes in the metabolites ornithine and cystine (Palmer et al., 2013). Changes in these metabolites are measured in response to treatment and then used in a ratio (o/c ratio) across an 8-point dose response curve. The developmental toxicity potential (dTP) is the interpolated exposure level (concentration) of a test article where the dose response curve crosses a defined developmental toxicity threshold (dTT). Exposure levels greater than this concentration are associated with developmental toxicity. The assay was 85% accurate in predicting the developmental toxicity potential of 80 compounds with a broad range of chemotypes (89% specificity, 82% sensitivity). The data generated with the devTOXqP assay presents an opportunity to include an *in vitro* human endpoint in read-across or weight of evidence approaches.

A set of five structurally similar triazole fungicides (flusilazole, hexaconazole, propiconazole, triadimefon and myclobutanil) were evaluated in the devTOXqP assay to demonstrate how the assay can be used to strengthen read-across and weight of evidences approaches (Tab. 1). Myclobutanil was used as an example compound with an uncertain developmental toxicity profile. Flusilazole was the most potent chemical *in vivo* and in the devTOXqP assay, with a developmental toxicity potential at 17 μM (Tab. 2). Myclobutanil had a developmental toxicity potential similar to its analogs and was classified as a developmental toxicant, which is consistent with published *in vivo* data (Tab. 2). Additionally, myclobutanil had the highest NOAEL for developmental toxicity *in vivo*, which is consistent with it being the least potent in the devTOXqP assay. Taken as part of a weight of evidence approach, this human data point would help to define potential for risk.

### 2.2 Using big data to establish chemical profile for biological similarity read-across studies

#### 2.2.1 Available sources of biological data

The term “big data” describes a collection of data sets that are so large and complex that the data is too difficult to process by traditional data analysis tools. Modern toxicity research has moved into the big data era as massive biological data for compounds of interest (e.g. toxicants) became available (Zhu et al., 2014). There are two major sources of biological data: One biological data source is HTS of large libraries of compounds in toxicity studies. There has been a huge increase in the number of compounds and associated testing data in different *in vitro* screenings. Besides that, there are also efforts to curate historical *in vivo* toxicity data to share with the public. Table 3 shows some examples of these data collections distributed through various data sharing programs. PubChem is a public repository for chemical structures and their biological properties (Wang et al., 2009, 2010). Bioactivity data in PubChem were contributed by hundreds of institutes, research laboratories, and specifically those screening centers under the NIH Molecular Libraries Program (MLP) (Austin et al., 2004). For example, the NIH Chemical Genomics Center (NCGC) was created in 2005 as a comprehensive Screening Center in the NIH MLP (Thomas et al., 2009). The mission of the NCGC is to apply the tools of small molecule screening and discovery to toxicology studies. Every year the NCGC generates millions of toxicity bioassay data points by testing thousands of diverse compounds and shares all the data with the research community via PubChem. The unique quantitative High Throughput Screening (qHTS) technique developed and optimized by the NCGC generates data in high quality and standardized form (Inglese et al., 2006). Another large reservoir of toxicity bioassay data in PubChem comes from the European Bioinformatics Institute (EBI) (ChEMBL^3^). The EBI’s goal is to provide freely available data and bioinformatics services to all parts of the scientific community. As a part of this goal, the ChEMBL database was constructed for screening data of both chemical toxicity and Absorption, Distribution, Metabolism and Excretion (ADME) properties. ChEMBL version 11 (ChEMBL_11) was launched in 2011. It includes 3.3 million bioassay measurements covering 629,943 compounds (Gaulton et al., 2012). This was obtained from curating over 42,500 scientific publications.

The ToxCast program of the US EPA was initiated to find alternatives to animal models (Reif et al., 2010; Dix et al., 2007). For this purpose, this program intentionally tested compounds with rich animal toxicity information, which generate a database containing both *in vitro* and *in vivo* toxicity data. Currently the ToxCast data, along with animal toxicity data, is shared via Aggregated Computational Toxicology Resource (ACToR^4^) (Judson et al., 2008, 2012) portal. Similarly, but different compared to ACToR, ToxNET^5^ contains and allows navigation through 16 separate databases of much more diverse chemicals (Fonger et al., 2000). ToxNET was developed by the National Library of Medicines’ (NLM) Division of Specialized Information Services (SIS). By grouping the databases together, ToxNET allows for all information to be accessed from one query form. Although there are 14 separate databases, some are very similar and are grouped together in the example report.

In response to the shortage of alternative testing methods, the European Commission and the European Cosmetics Trade Association, Cosmetics Europe, launched over the last five years the research initiative called Safety Evaluation Ultimately Replacing Animal Testing (SEURAT-1^6^) in 2011 (Vinken et al. 2012). It is called “SEURAT-1”, indicating that more steps have to be taken before the ultimate goal of full animal replacement will be reached. Under the SEURAT-1 initiative, there were five research projects and one coordinating project funded and extensive data curation/management work was involved (Kohonen et al. 2013). For example, one of these projects, the COSMOS project, was dedicated to the development of freely available tools and workflows to predict the safety of cosmetic ingredients to humans (Yang et al., 2013). In the released COSMOS database web portal^7^, there are over 5,500 unique cosmetic-relevant compounds with their respective *in vivo* toxicity data. A similar effort is the recent curation of REACH toxicity data (Hartung, 2010; Hengstler et al., 2006) from the publicly available registration summary data (Luechtefeld et al., 2016a–d, this issue).

Another rapidly growing area of interdisciplinary research generating big data is toxicogenomics (TGx), which aims to study the underlying molecular mechanisms of toxicity and address challenges that are difficult to overcome by conventional toxicology methods by integrating genomic technology with bioinformatics. Toxicogenomics is a field of toxicology that addresses information concerning gene expression changes, and in extension also protein, and metabolite changes (Bouhifd et al., 2013; Ramirez et al., 2013), within a particular cell or tissue of an organism in response to chemicals. It has to be noted that transcriptomics is certainly most advanced among the omics technologies (van Vliet, 2011) with regard to standardization and quality assurance, but other omics technologies such as metabolomics are catching up (Bouhifd et al., 2015). Many modern in vitro toxicity studies now address relevant toxicity mechanisms and these findings can be translated into biomarkers that could be applied to human exposure studies (McHale et al., 2010; Blaauboer et al., 2012).Several extensive publicly available TGx databases based on good experimental designs, such as the Japanese Toxicogenomics Project (TGP) (Uehara et, al. 2010) and PredTox (Suter, et. al, 2011), provide enormous opportunities to evaluate and investigate a large set of TGx assays systematically, which gives a landscape of TGx and more objective understanding of toxicity mechanisms. TGx investigations generate enormous amounts of “omics” data that are meant to predict toxicity or genetic susceptibility induced by chemicals. The Chemical Effects in Biological Systems (CEBS^8^) database developed by the NIEHS is now the public repository for all NTP conventional toxicology and carcinogenicity data as well as NCGC HTS data (Waters et al., 2008) along with the Comparative Toxicogenomics Database (CTD^9^) at Mount Desert Island Biological Laboratory aims to promote comparative studies of genes and proteins across species (Mattingly et al., 2006a,b; Mattingly et al., 2004; Mattingly et al., 2003). CTD data is searchable through the ToxNET portal. Similar efforts in toxicogenomics data curation, but with more specific research goal, are DrugMatrix^10^ and Cmap^11^ (Lamb et al., 2006).

#### 2.2.2 Evaluating biological similarity based on big data

Although the read-across based on the hypothesis that similar structures have similar toxicological profiles, usually the information derived from the chemical structure is limited. Therefore, information regarding the biological properties of the chemicals, both target and analogs, is the key support to the read-across. The biological similarity refers to the similar results from one or more assays for two chemicals. One of the approaches is to use the results from a large number of assays, usually high throughput assays, to profile the biological fingerprint of a chemical (Kim et al., 2016; Zhang et al., 2014; Sipes et al., 2013). If two chemicals have similar bioprofiles, they will be considered to be biologically similar (Low et al., 2013; Zhang et al., 2014). However, it is not easy to apply this in any real case of read-across. First, it requires comprehensive information from toxicogenomics studies and/or high-throughput assays for both target and analog. It is unusual that both target and analog have been tested in the same toxicogenomics studies and/or high-throughput assays. If we generate the data required as needed, the costs may not be much less than just testing the chemical of interest for any specific toxicity endpoint. Second, irrelevant information might be included when conducting read-across for a specific toxicity endpoint. For example, if using read-across to fill the data gap for skin sensitization, endocrine system related *in vitro* assays are not relevant to this endpoint. Therefore, one should be cautious using a universal bioprofile to evaluate the biological similarity to support an endpoint specific read-across. Another approach is based on the understanding of the mechanism of the specific toxicity, which is using one or a few closely related bioassays to compare the biological similarity. For example, one can use the Direct Peptide Reactivity Assay (DPRA) and KeratinoSens assays to profile the biological similarity for skin sensitization, or use the bluescreen assay to profile the biological similarity for genotoxicity. Assays that map to the estrogen receptor pathway can be used to define biological similarity for potential endocrine disrupting compounds. It is worth mentioning that biological similarity should serve as a weight of evidence to evaluate the read-across, but structural similarity will be usually the first tier for similarity criteria.

**Fig. 1:**
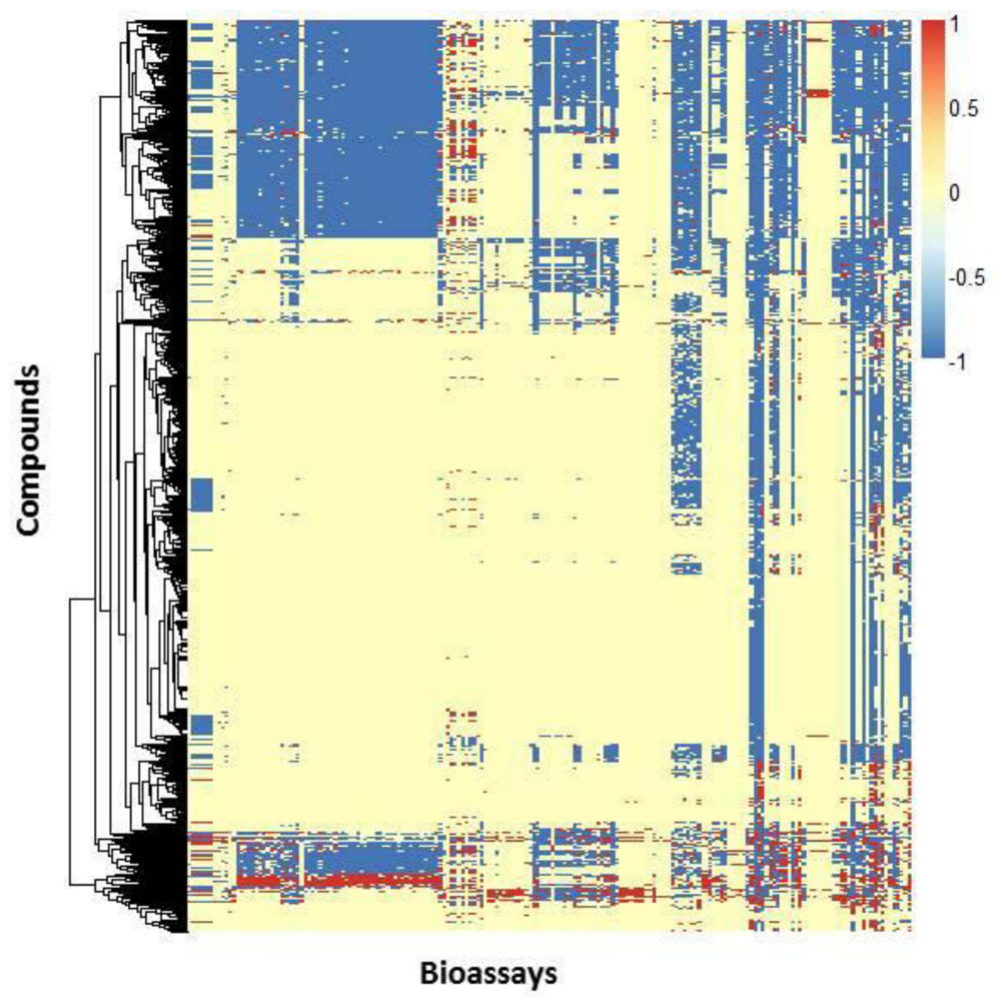
The response space of 962 ToxCast compounds represented by the data obtained from 193 PubChem bioassays The red dots represent active responses; the blue dots represent inactive responses, and the yellow dots represent no available testing data or inconclusive results.

In the current big data era, the bioassay response profile can be very large for some compounds (e.g. well-known toxicants) (Zhu et al. 2014). If all the public data for these compounds are used to create a profile, the initial profile can be large, complex and unorganized. For example, Figure 1 shows the PubChem response space of 962 ToxCast compounds by using 193 PubChem assays (accessed August 2013). So the public resources shown in Table 3 contain lots of biological data that will be useful for read-across purposes.

It is understandable that most areas within the initial response map are either “no testing” or “inconclusive” because many bioassays have only been applied to a small portion of this large chemical set. Furthermore, the nature of HTS assays, many of which represent specific interactions, results in a biased distribution of responses for the target chemicals (many more “inactives” than “active” data entries). Since not all the bioassay data are relevant or useful for a particular type of toxicity, in the big data scenario, the most critical issue is to identify useful *in vitro* data. In principle, this could be done by a human expert using the knowledge of the design and quality of each particular bioassay (e.g., the “Confidence Score” assigned during manual curation to each assay in ChEMBL). However, in the big data era, the data-driven approaches should be developed preferably by fully automatic techniques. We recently developed an automatic bioassay system to evaluate and extract the relevant bioassay data based on the *in vitro-in vivo* relationship (Zhang et al., 2014; Wang et al., 2015; Kim et al., 2014). Using this approach, we analyzed the current REACH compounds with their rat oral acute toxicity. Table 4 shows three REACH compounds with their chemical nearest neighbors in the same set. It is obvious that these three pairs of chemicals’ nearest neighbors have quite different acute toxicity results. Any read-across approaches only based on molecular structures will not be able to differentiate them. Therefore, these activity cliffs in the REACH data set will prompt prediction errors of any QSAR models.

We can integrate public bioassay results for these compounds as extra information for read-across purposes. If searching the PubChem portal using an in-house profiling tool, hundreds of PubChem assays containing experimental data for REACH compounds were automatically extracted (Luechtefeld et al., 2016a, this issue). These experimental biological data can be viewed as extra descriptors and a similarity search can be applied by using these data as bioprofiles. This way, the biological nearest neighbors for the target three compounds can be found within the REACH data set, as shown as the third compound in each group of Table 4. It is clear that the biological nearest neighbors have much more similar acute toxicity results to the three target compounds when comparing to the chemical nearest neighbors, indicating the value of using these extra biological data in the read-across procedure. This effort provides a potential solution to the pitfall of applying QSAR as read-across approaches induced by the activity cliff issue.

#### 2.2.3 Case study: using complex high-throughput biological data to support read-across - BioActivity-based read-across (BaBRA) using ToxCast Data

As highlighted above, the advent of high-throughput screening and research initiatives such as Tox21 and ToxCast provide data on a range of targets and pathways that may be linked to toxicity (Judson et al., 2010; Betts, 2013). The Tox21 program has screened over 8,000 chemicals in approximately 60 assays, and ToxCast testing includes a much broader range of assays, with around 800 targets, on a fewer number of chemicals (~2,000). The ToxCast dataset^12^ in particular affords a unique opportunity to attempt BioActivity Based Read-Across (BaBRA), due to the wide coverage of biological space and range of assays from different cell types, species, and technology platforms. A number of predictive models have identified critical pathways, such as embryonic vascular development, and characterized similar chemical activity against the identified molecular targets as a way to prioritize chemicals for their potential to cause toxicity, e.g. developmental defects (Kleinstreuer et al., 2011; Knudsen and Kleinstreuer, 2012). Supervised analyses such as Support Vector Machines (SVM) have successfully predicted mechanisms such as phosphodiesterase inhibition and glucocorticoid receptor agonism for unknown chemicals based on similar protein expression profiles in primary human cells (Kleinstreuer et al., 2014). Others have described an approach that is pathway-agnostic and more closely resembles traditional structure-based read-across, with the addition of all available *in vitro* assay data as features to determine biological similarity (Low et al., 2013; Kim et al., 2016). Here we examined analogous approaches, both encompassing and pathway-specific, with a novel mathematical definition of similarity that used *in vitro* bioactivity data from ToxCast, as well as structural features to classify different adverse effects *in vivo*.

**Fig. 2:**
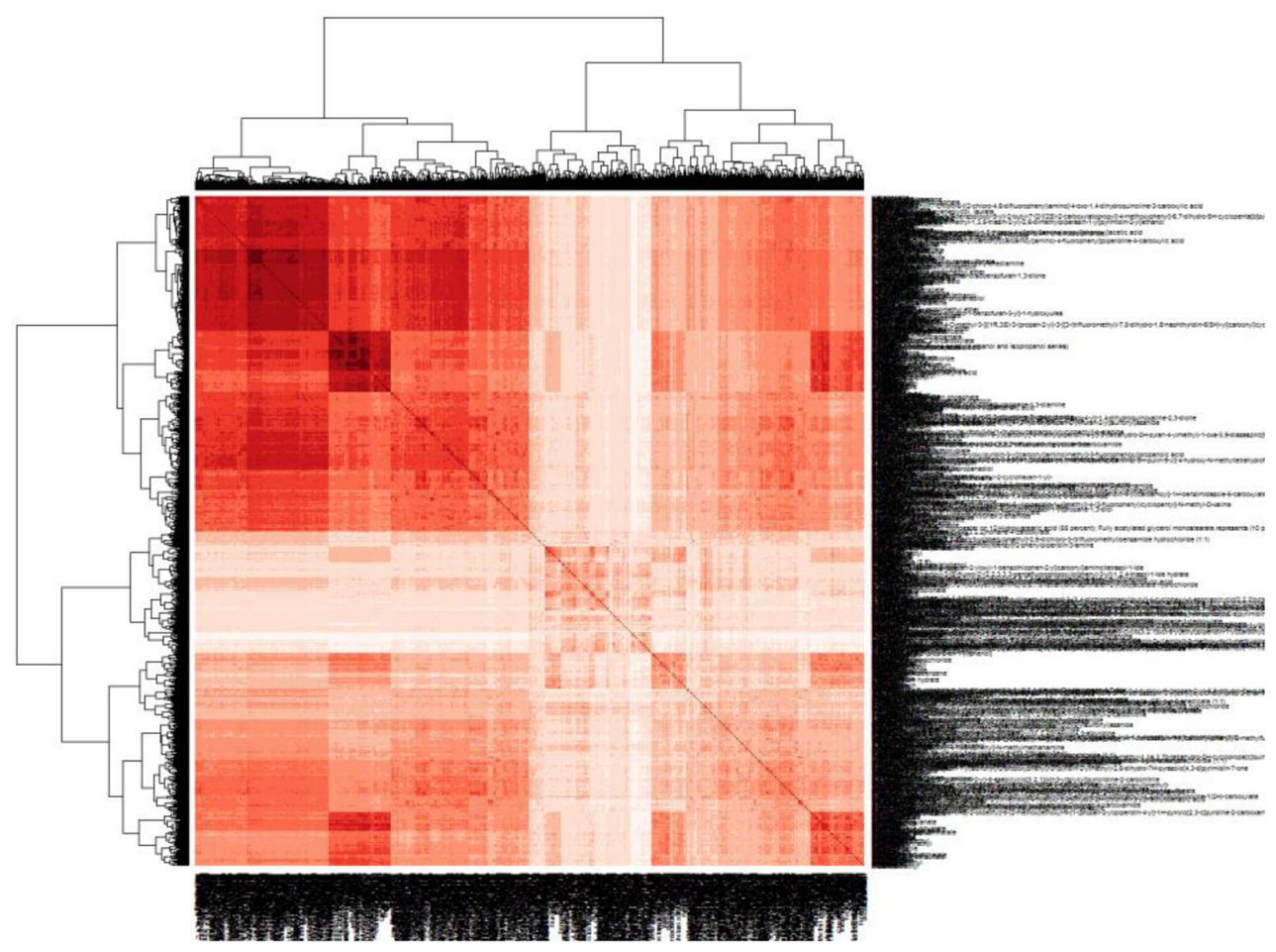
Random Forest Proximity Matrix for 1,056 ToxCast Phase I/II chemicals Chemicals are clustered based on their similarity across all 800 ToxCast *in vitro* assay targets. Chemical ordering is the same on each axis, and the unity correlation is shown along the diagonal. Darker red coloring indicates a higher degree of similarity.

Here, ToxCast *in vitro* assay data was used to perform BaBRA to predict *in vivo* endpoint information for one chemical by using data from the same *in vivo* endpoint from another chemical, which had similar *in vitro* activity. This biological databased similarity was also enriched with structural similarity (St.BaBRA) and used to make predictions for a chemical’s *in vivo* toxicity based on its nearest neighbors. The measure of chemical similarity was calculated using an unsupervised random forest approach to produce a proximity matrix. Briefly, a random forest is a collection of tree predictors such that each tree depends on the values of an independently sampled random vector from within the feature space (i.e. *in vitro* assay data and/or structural descriptors). When random forest is run in an unsupervised fashion to calculate the proximity matrix, the original data is considered as class 1 and a synthetic second class of the same size is created by sampling at random from the univariate distributions of the original data and labeled as class 2. In this way class 2 maintains the distributions of the variables but destroys the dependency structure in the original data. The N×N proximity matrix is formed by growing a large number of trees (here 10,000) based on the artificial two-class problem, and for each tree if chemicals x and y end in the same terminal node their proximity increases by one. Finally, the proximity scores are normalized by dividing by the number of trees. Figure 2 shows the proximity matrix for the ToxCast Phase I and II chemical library, based on the entire set of *in vitro* assays.

Several proximity matrices were calculated to characterize similar bioactivity across the ToxCast chemical library, based on all the *in vitro* assays (Figure 2), on a subset that were run in concentration response (excluding those that were only run in a single concentration screen), or on assays that mapped to a particular biological pathway relevant to the endpoint of concern. These proximities, with and without enrichment for structural similarity, were used to make predictions for a particular chemical based on the *in vivo* outcomes observed in *k*-Nearest Neighbor (*k*NN) space. The following equations define the predicted activity for a chemical against a toxicity endpoint based solely on *in vitro* biological similarity,

$$A_{\mathrm{pred}}=\frac{\sum_{i=1}^{k}P_{i}*A_{i}}{\sum_{i=1}^{k}P_{i}}$$

or with the inclusion of structural similarity,

$$A_{\mathrm{pred}}=\frac{\sum_{i=1}^{k}P_{i}*A_{i}+\sum_{i=1}^{k}S_{i}*A_{i}}{\sum_{i=1}^{k}P_{i}+\sum_{i=1}^{k}S_{i}}$$

where *A_pred_* is the predicted activity, *k* is the number of nearest neighbors, *P_i_* is the proximity score based on the ToxCast data, and *S_i_* is the structural similarity score based on the Tanimoto index (Abdo and Salim, 2009). The BaBRA and St.BaBRA predictions were produced based on a variety of proximity matrices, as mentioned previously, and compared to a range of *in vivo* endpoints from the Toxicological Reference Database (ToxRefDB^13^) and from a database of guideline-like uterotrophic studies curated by the National Toxicology Program Interagency Center for Evaluation of Alternative Toxicological Methods (NICEATM^14^).

Multiple study types are represented in ToxRefDB, namely prenatal developmental, multigenerational reproductive, subchronic, and chronic cancer studies, with corresponding lowest effect levels (LELs) on a per-chemical basis for a hierarchy of apical endpoints (e.g. skeletal malformations, litter size, liver tumors, etc.). Many of these endpoints are highly unbalanced, with either positive or negative results significantly overrepresented. To deal with the biased data, random sampling from the positive/negative space was used to create a balanced dataset for each ToxRefDB endpoint. Parameter sweeps were used to define the optimal values for the respective *k*NN space and apply a minimum threshold for the similarity scores. Endpoints from all four study types were predicted using the BaBRA and St.BaBRA approaches, and showed generally poor predictive performance. The best BaBRA model for a ToxRefDB endpoint used a proximity matrix based on the ToxCast assays that were run in concentration response to predict the existence of a LEL for reproductive impairment. This model was based on 256 compounds (even distribution between positives and negatives), an optimal *k*NN space of 6 nearest neighbors, and achieved sensitivity, specificity and balanced accuracy of 70% (p-value < 1×e^−6^). The addition of structural similarity did not improve the model. Interestingly, this level of predictivity may actually reflect the degree of variability in the *in vivo* endpoint being predicted and/or the presence of multiple mechanisms contributing to an observed endpoint.

To create BaBRA/St.BaBRA frameworks that were informed by biological relevance and anchored to highly curated data, we used only assays that mapped to the estrogen receptor (ER) pathway to create a proximity matrix, and attempted to predict the outcome in high quality uterotrophic studies that specifically measure an estrogenic response *in vivo*. The NICEATM uterotrophic database (Kleinstreuer et al., 2015) contains results from >700 studies whose protocols were evaluated based on adherence to a set of minimum criteria from internationally harmonized test guidelines from the U.S. EPA and the Organization for Economic Cooperation and Development (OECD). The best model performance was achieved using a proximity matrix calculated with only the 18 ToxCast assays that map to the ER pathway, with an optimal *k*NN of 3 nearest neighbors, for compounds with uterotrophic studies that met all the minimum criteria to be considered guideline-like and had reproducible results across multiple labs. Both BaBRA and St.BaBRA approaches resulted in a sensitivity of 95%, a specificity of 98%, and a balanced accuracy of 97% (p-value < 1×e^−15^), with dibutyl phthalate as the only false positive and octylmethyltetrasiloxane (D4, a highly volatile compound) as the only false negative.

BaBRA and St.BaBRA are approaches that show great promise within certain applicability domains and well-curated data sets. However, broad *in vitro* activity patterns across a wide range of assays are difficult to correlate with apical *in vivo* toxicity endpoints, even when enriched with structural similarities. Feature selection and optimization methods should be explored to improve predictive accuracy and applicability. For example, identifying features that provide the best separation between positive and negative space for each ToxRefDB endpoint in combination with *in vivo* data curation (e.g. lowest adverse effect levels instead of LOELs, study quality evaluation) will improve the applicability of read-across. Further, biological pathway knowledge can be used to define the assay/proximity space that is relevant to the endpoint of interest (e.g. endocrine targets to predict reproductive impairment, or cancer hallmarks to predict carcinogenesis).

### 2.3 Using omics data for establishing biological similarity for read-across

Grouping of chemicals based on structural relationships should also be complemented by omics data: The advantage can be illustrated by two examples: 1) 2-Acetylaminofluorene (2-AAF) and 4-Acetylaminofluorene (4-AAF) are structurally very similar. However, the toxicological profile of these two compounds differs significantly. 2-AAF is a strong liver enzyme inducer, leading in long term studies to liver tumors, whereas 4-AAF only slightly induces liver enzymes and does not induce the formation of liver tumors. This is reflected in different metabolome changes induced by these two compounds in rat plasma (van Ravenzwaay et al., 2012); 2) A lot of compounds with different structures (e.g., fibrates, phthalates, perfluorinated fatty acids) are stimulating the peroxisome proliferator activated receptor alpha (PPARα) leading to hepatomegaly and liver tumors in rodents (Youssef, 1998). These compounds can be grouped by typical metabolite changes in rat plasma in one class, and can be differentiated from other liver tumor-inducing compounds, e.g. liver enzyme inducers (van Ravenzwaay et al., 2010a).

A prerequisite for using omics data for read-across is a standardized technique and a database with reference compounds for application of grouping with data-poor chemicals. Regarding metabolomics, BASF and Metanomics have established a standardized technology (Looser et al., 2005) and built up such a database (MetaMap^®^ Tox) with about 600 compounds administered to rats in repeated dose studies (van Ravenzwaay et al., 2015). The toxicological activity of data-poor chemicals in rats can be assessed by a standardized evaluation procedure with this database: 1) Profile strength: it is assessed if the number of metabolite changes in rat plasma is above a threshold representing a treatment-related effect (van Ravenzwaay et al., 2014); 2) Pattern ranking: metabolomics mode of action patterns are defined with reference compounds representing a unique set of metabolites changed in the same way. The fit of the metabolome of new compounds to these patterns is evaluated statistically and by toxicological expert judgement; 3) Treatment correlation: The measured metabolome of data-poor chemicals is compared with reference compounds in the database by correlation statistics; 4) Pathway analysis: eventually, endogen metabolite changes can explain or monitor key events in the adverse outcome pathway (e.g., accumulation of tyrosine and 4-hydroxyphenylpyruvate of 4-hydroxyphenylpyruvat dioxygenase inhibitors, i.e. a herbicide compound class). As result of the mentioned evaluation process, an assessment can be made regarding: 1) target organ; 2) systemic toxicity mode of actions by comparison with reference compounds; 3) which pathways or which chemical groups of metabolites (e.g. aromatic amino acids, unsaturated long chain fatty acids etc.) in the rat physiology are affected. For example, differentiating direct thyroid hormone synthesis inhibitors from compounds accelerates the thyroid hormone clearance (Montoya et al., 2014). The assessment is restricted to the set of reference compounds in the database and the established metabolite patterns, defining, which mode of actions can be covered.

To increase confidence in the results, different levels of validation procedures have to be performed. Apart from the technical validation of the applied methods and the statistics, influencing factors and the variation of the biological system (here metabolomics in rat plasma) have to be assessed. Regarding the MetaMap^®^ Tox database several aspects have been published, such as influence of rat strains (Strauss et al., 2009), influence of the diet (Mellert et al., 2011), reproducibility and robustness of the biological system (Kamp et al., 2012).

Concerning read-across for the absence of a toxic effect, a quantitative risk assessment for the regarded endpoints is necessary. The no adverse effect level (NOAEL) can be determined with omics technologies as the absence of a consistent pattern of change associated with an adverse effect (ECETOC, 2008, 2010, 2013). This is assessed for metabolomics by the fit of the metabolite profile of new compounds to the established adverse mode of action patterns in the MetaMap Tox database (van Ravenzwaay et al., 2014). There are some publications comparing transcriptomic/metabolomic data and most sensitive traditional toxicity data regarding the benchmark dose or NOAEL dose, stating that the sensitivity of the omics technologies compared to “traditional” toxicity measurements (histopathology, clinical pathology) are in a comparable magnitude order (van Ravenzwaay et al., 2014, Thomas et al., 2011, 2012).

## 3 Discussion

The above sections and examples together here show that the concept of biological similarity enhances read-across: if the target of interest and the similar compounds have been tested in the same set of high-throughput assays, one can use a bioprofile (i.e. a collective set of results from different assays) to profile the target compounds against the tested compounds, and then compare the bioprofile between them. The key of this procedure is to prove the selected assays are related to the toxicological endpoint of interest, either from the understanding of the toxicological mechanism (e.g. as characterized by an AOP) or from the correlative data analysis (e.g. significant relationship between the bioprofile and the toxicological effect). If there is any data gap for generating the bioprofile (i.e. lacking information for certain *in vitro* assays,) one might use QSAR models to predict the results of the *in vitro* assay. When applying QSAR modeling, one should follow the respective OECD guidance for QSAR (Gramatica, 2007).

The increasing availability of biological data via the data sharing depositories will augment such support of read-across and grouping by big data. The curation of such datasets and the respective data-sharing by companies, organizations and individual researchers needs to be further encouraged and possibly furthered with some incentives. Alternatively, wholesome profiling, typically by transcriptomics or metabolomics, of the biological effect of substances in complex systems representing many targets for perturbation can allow an individual assay to support similarity arguments.

## 4 Conclusions

Taken together, the approaches presented here cannot yet be considered as standardized tools for read-across. However, they promise already now on a case-by-case basis to support read-across considerations and should be considered when the respective test data are available or can be obtained with reasonable efforts. For the future, more accessible standardized testing environments might offer bioprofiling of substances and thereby open the doors for enhanced read-across of substances, which have not been broadly studied in the scientific literature.

## Figures and Tables

**Tab. 1:** General information and properties of the analogs

Property	Flusilazole	Hexaconazole	Propiconazole	Triadimefon	Myclobutanil
**Use**	Fungicide/antibacterial drug	Fungicide	Fungicide	Fungicide	Fungicide
**Structural representation**	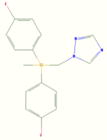	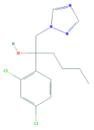	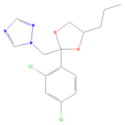	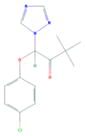	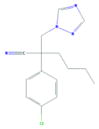
**CASRN**	85509-19-9	79983-71-4	60207-90-1	43121-43-3	88671-89-0
**Molecular weight (g/mol)**	315.3927	313.0749	341.0698	293.0931	288.1142
**Physical state at 20°C & 101.3 kPa**	Solid	Solid	Liquid	Solid	Solid
**Water solubility (mg/L) at 20 or 25°C**	43	1.29	100	71.5	142
**log P (octanol–water)**	4.68	3.7	3.7	2.8	2.9

**Tab. 2:** Summary of test article ADME and toxicity data

	Flusilazole [1]	Hexaconazole [2]	Propiconazole [3]	Triadimefon [4]	Myclobutanil [5]
**ADME Properties**					
***In vivo* half-life (plasma/serum)**	NA	NA	24–31 hr	~4 hr	Biphasic Rapid Phase: 5.25 Slow Phase: 25.7
**Rate and extent of oral absorption**	Rapid & Extensive (up to 80%)	NA	>80% in 48h	28% in females, 67% in males as urinary excretion	Rapidly absorbed (> 89%)
**Distribution**	Widely	Widely distributed; highest concentrations in liver, intestinal tract and adrenal cortex	Widely distributed; highest concentrations in the liver and kidney	Widely distributed in kidneys and liver	Widely distributed
***In vivo* metabolism**	Extensive	Extensive	Extensive	Rapid & Extensive	Rapid & Extensive
**Most active CYPs**	NA	NA	NA	CYP2C and CYP3A	CYP2C and CYP3A
Excretion*	96 hr	72 hr	24 hr	96 hr	96 hrs
**Primary route of excretion**	urine	43% urine/53% feces (m) 66% urine/29% feces (f)	Even distribution in urine & feces	Feces (m) urine (f)	Even distribution in urine & feces
**Toxicological Data**					
**Acute toxicity, oral, LD50 (mg/kg bw)**	674	2189	1517	363–1855	1600
**Genotoxicity**	Negative	Negative	Negative	Negative	Negative
**Short-term Toxicity Studies**					
**Target**	Liver and Urinary bladder	Liver	Body weight, liver, erythrocytes	Liver	Liver
**Oral NOAEL (mg/kg bw per day)**	9	2.5	76	150	51.5
***In Vivo* Developmental Toxicity Studies**					
**Target/critical effect**	Skeletal anomalies, malformations at higher doses	Fetal Toxicity, skeletal variations	Skeletal variations	Skeletal variations	Fetal toxicity/increased number of early resorptions and lower fetal weights
Developmental toxicity NOAEL** **(mg/kg bw per day)**	2	2.5	30	30	93.8
**Maternal toxicity NOAEL (mg/kg bw per day)**	10	25	90	10	93.8
**devTOX*^qP^* Results**					
**dTP (μM)**	**17**	**22**	**26**	**35**	**51**

**Note:** NA: Data not available. *In vivo* data summarized from rat studies.

* Excretion is greater than or equal to 90% of radiolabel.

** Developmental toxicity includes embryo/fetal toxicity and teratogenicity.

**[1]** Adcock and Tasheva, 2009

**[2]** Tonkelaar and van Koten-Vermeulen, 1991

**[3]** Dewhurst and Dellarco, 2006

**[4]** Zarn et al., 2006

**[5]** Yoshida and McGregor, 2015

**Tab. 3:** Public databases of toxicity data

Name	General Information	Data description
PubChem	Over 50 million compounds, over 700,000 bioassays, over 13 billion data points	Toxicity, genomics and literature data
ChEMBL	Over 600,000 compounds, 3.3 million bioassay readout data	Literature toxicity data
ACToR	The toxicity results from 100 various data resources	Both in vitro and in vivo toxicity data
ToxNET	Over 50,000 environmental compounds from 16 different resources	Both in vitro and in vivo toxicity data
SEURAT	Over 5,500 cosmetic-type compounds in the current COSMOS database web portal	Animal toxicity data
REACH	816,048 studies for 9,800 substances and 3,600 study types	Data submitted in EU chemical legislation, made machine-readable by Luechtefeld et al. 2016a (this issue)
CTD	Over 13,000 compounds, over 32,000 genes, over 6000 diseases	Compound, gene and disease relationships
CEBS	About 10,000 toxicity bioassays from various sources	Gene expression data
DrugMatrix	About 600 drug molecules and 10,000 genes	Gene expression data
Cmap	About 1,300 compounds and 7,000 genes	Gene expression data

**Tab. 4:** Three REACH compounds (the first compound) with their chemical nearest neighbor (the second compound) and biological nearest neighbor (the third compound)

	Compounds	LD_50_ (mg/kg)	Bioprofiles*
1	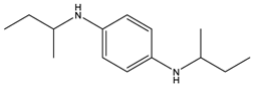	181	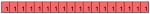
	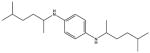	730	N/A**
	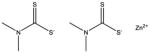	320	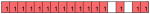
2	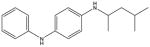	949	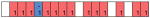
	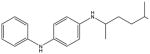	2,100	N/A
	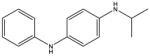	520	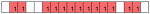
3	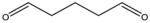	206	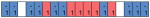
	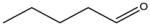	6,490	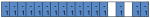
	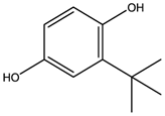	1,041	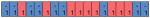

* The bioprofile consists of 18 PubChem assays (PubChem assay AIDs 427, 542, 544, 545, 546, 921, 963, 964, 966, 968, 973, 974, 993, 504832, 651802, 686979, 743041, 743086) which were selected for calculation since they contain the largest number of active responses per assay in REACH compounds. The red color indicates active response, blue color indicates inactive response and white color indicates no data available.

** N/A indicates there is no data available for this compound within these assays.

## Footnotes

2 WEC: whole embryo culture; mEST: mouse embryonic stem cell test; ReProGlo: Wnt signaling EST (Hecker, et. al, 2011); Ishikawa test: mRNA levels of progesterone receptor; AR: Androgen Receptor; ER: Estrogen receptor.

3 www.ebi.ac.uk/chembldb/index.php

4 http://actor.epa.gov/actor

5 http://toxnet.nlm.nih.gov

6 www.seurat-1.eu

7 http://cosmosdb.cosmostox.eu

8 http://tools.niehs.nih.gov/cebs3/ui/

9 http://www.niehs.nih.gov/research/resources/databases/cebs/index.cfm

10 https://ntp.niehs.nih.gov/drugmatrix/index.html

11 https://www.broadinstitute.org/cmap/

12 http://actor.epa.gov/actor/toxcastdbata.jsp

13 http://actor.epa.gov/toxrefdb

14 http://ntp.niehs.nih.gov/pubhealth/evalatm/tox21-support/endocrine-disruptors/edhts.html
